# Telemedicine in total joint arthroplasty: toward continuous and patient-centered postoperative care

**DOI:** 10.3389/fmed.2026.1781071

**Published:** 2026-07-08

**Authors:** Damei Zhang, Qin Zhong, Rong Zhou, Caixia Peng, Wenwen Huang, Chengyou Xie, Hui Xie, Shouyong Hu, Jie Zou

**Affiliations:** 1 Department of Orthopedics, Jingmen Central Hospital, Jingmen Central Hospital Affiliated to Jingchu University of Technology, Jingmen, China; 2 School of Medicine, China Three Gorges University, Yichang, China

**Keywords:** postoperative care, telemedicine, telerehabilitation, total hip arthroplasty, total knee arthroplasty

## Abstract

Telemedicine has increasingly become an important component of orthopedic postoperative care, particularly in total joint arthroplasty (TJA). This mini review synthesizes current evidence suggesting that specific telemedicine modalities–such as video-based consultations, smartphone-assisted wound monitoring, and structured telerehabilitation–may achieve comparable outcomes in terms of wound assessment accuracy, short-term functional recovery, and patient satisfaction in selected patients undergoing uncomplicated total hip or knee arthroplasty. By reducing geographical and logistical barriers, it enables more continuous and accessible follow-up, potentially supporting patient-centered recovery. Beyond clinical outcomes, telemedicine may facilitate multidisciplinary collaboration, support early identification of complications, and enhance psychosocial engagement through sustained patient–provider communication. Nevertheless, the existing evidence is heterogeneous, and its generalizability is limited by factors such as digital access disparities, variability in study design, and evolving regulatory frameworks. Future developments, including artificial intelligence and wearable technologies, may further expand the role of telemedicine in postoperative care. Overall, telemedicine represents a promising adjunct to conventional follow-up, although its optimal integration into routine orthopedic practice requires further validation.

## 1 Introduction

Telemedicine has transitioned from an emerging concept to an integral component of modern healthcare systems–a shift markedly accelerated by the COVID-19 pandemic (1, 2). Its application in managing postoperative pathways for elective surgeries such as total joint arthroplasty (TJA) is particularly pertinent. Successful recovery after TJA depends heavily on sustained, high-quality rehabilitation and consistent follow-up. However, conventional in-person models are frequently challenged by patient-level barriers–geographical distance from specialist centers, limited mobility during early recovery, and the socioeconomic burden of repeated clinical visits. These barriers often result in fragmented care, poor rehabilitation adherence, and suboptimal functional outcomes.

For patients undergoing TJA, telemedicine may offer a promising approach to supporting continuity of care and enabling a personalized rehabilitation journey. Structured remote monitoring and virtual follow-ups preserve the therapeutic connection between patient and surgeon, improving safety and adherence through timely interventions (3). Furthermore, digital platforms integrating wearable sensors and mobile applications provide real-time feedback and tailored exercise prescriptions that optimize functional recovery (4). A growing body of evidence suggests that this integrated, technology-enabled approach may achieve comparable outcomes–particularly in terms of patient satisfaction, adherence, and early functional recovery–when applied to structured telemedicine interventions in patients undergoing uncomplicated primary TJA (5–7).

This mini review aims to provide a focused synthesis of current evidence on the role of telemedicine in postoperative care following TJA, with particular emphasis on remote monitoring, diagnostic reliability, telerehabilitation, and interdisciplinary care integration. In this review, telemedicine modalities refer primarily to video-based consultations, smartphone-assisted wound monitoring, remote patient monitoring using wearable or app-based tools, and structured telerehabilitation programs delivered through digital platforms. Rather than conducting a formal systematic review, we adopted a narrative approach to summarize representative and high-impact studies in this rapidly evolving field. The literature included in this review was selected based on relevance to postoperative TJA care, methodological rigor (including randomized trials, cohort studies, and meta-analyses), and recency, with priority given to studies published in peer-reviewed journals within the past decade. By delineating key domains of telemedicine application, this review seeks to clarify its clinical value, identify existing limitations, and outline future directions for equitable and patient-centered orthopedic care.

## 2 Literature selection

This mini review was conducted using a narrative, non-systematic approach. Relevant literature was identified through searches of major medical databases, including PubMed, Web of Science, and Scopus. Keywords included “telemedicine,” “total hip arthroplasty,” “total knee arthroplasty,” “postoperative care,” and “telerehabilitation.” Studies were included if they focused on telemedicine applications in postoperative management of total hip or knee arthroplasty. Priority was given to randomized controlled trials, cohort studies, systematic reviews, and meta-analyses published in English. Additional references were identified through manual screening of reference lists from key articles. While a formal systematic search strategy was not employed, efforts were made to include representative and high-quality studies to provide a balanced and comprehensive overview of the field. While this review prioritizes TJA-specific evidence, selected studies from broader orthopedic and general telemedicine literature were included where direct TJA data remain limited. Such studies were used to inform transferable mechanisms relevant to postoperative TJA care–including remote monitoring, rehabilitation adherence, interdisciplinary coordination, and psychosocial support–and were interpreted as supportive rather than primary evidence. Based on these selection principles, the principal studies informing this review are summarized in Table 1 to distinguish TJA-specific evidence from broader orthopedic or telemedicine literature.

**TABLE 1:** Principal studies informing telemedicine applications in postoperative TJA care.

References	Study design	Population	Telemedicine modality	Outcomes assessed	Key findings
Fahey et al. (1)	Systematic review	Orthopedic patients	Teleconsultation	Diagnostic concordance	Supports feasibility of remote assessment across orthopedic conditions
Kamecka et al. (8)	Methodological study	THA patients	Telemonitoring	Convalescence time, complication detection	Shorten the convalescence time and reduce the risk of complication
Marsh et al. (9)	Randomized controlled trial	TJA patients	Web-based follow-up	Cost-effectiveness, clinical outcomes	Comparable clinical outcomes with reduced need for in-person visits and potential cost benefits
Moore et al. (10)	Prospective cohort study	TKA/THA patients	Virtual follow-up	Patient satisfaction	High satisfaction rate, reflecting strong engagement and acceptance
Kuether et al. (11)	Retrospective cohort study	TJA patients	Telerehabilitation	Adherence, satisfaction	High patient satisfaction and improved compliance with rehabilitation
Windsor et al. (17)	Review	TJA patients	Teleconsultation	Diagnostic accuracy	Comparable outcomes to in-person visits
Bolam et al. (18)	Prospective cohort study	TKA patients	Wearable sensors	Range of motion, recovery tracking	Enabled real-time functional monitoring
Totty et al. (23)	Prospective cohort study	Clean/clean-contaminated vascular patients	Photo-based wound monitoring	Wound assessment	High diagnostic accuracy for wound complications
Moffet et al. (24)	Randomized controlled trial	TKA patients	Telerehabilitation	Pain, function, mobility	Comparable outcomes to conventional physiotherapy
Tousignant et al. (25)	Randomized controlled trial	TKA patients	Home-based telerehabilitation	Functional recovery	Equivalent clinical outcomes and patient satisfaction
Wang et al. (26)	Meta-analysis	Osteoarthritis patients	Telerehabilitation	Functional outcomes, adherence	Comparable effectiveness with improved adherence and potential cost benefits
Russell et al. (27)	Randomized controlled trial	TKA patients	Internet-based telerehabilitation	Functional recovery	Comparable outcomes to standard outpatient rehabilitation
Pastora-Bernal et al. (29)	Systematic review	TKA/THA patients	Telerehabilitation	Recovery	Strong evidence for recovery

### 2.1 Remote medical care in postoperative monitoring

The postoperative phase following TJA represents a period of heightened vulnerability, characterized by limited mobility, pain, and the need for vigilant wound surveillance. Traditional in-person follow-ups, while clinically valuable, impose substantial burdens on patients–particularly those living in rural areas or lacking reliable transportation. Telemedicine may help mitigate these challenges by reducing geographic and logistical barriers, thereby enabling continuous clinical oversight that transcends the conventional boundaries of hospital-based care (2).

A growing body of literature has substantiated these logistical and clinical benefits. Kamecka et al. (8) demonstrated that telemonitoring after total hip arthroplasty (THA) not only reduced travel-related burden but also minimized fragmentation of care, allowing clinicians to detect and address minor complications before they progressed to readmission-level events. Similarly, web-based follow-up systems in TJA have been shown to be feasible and effective, with no compromise in clinical outcomes while reducing the need for in-person visits (9). The significance of these findings extends beyond mere convenience–it suggests a shift toward more integrated and patient-centered recovery models, where virtual supervision ensures that postoperative monitoring becomes a sustained process rather than a series of episodic encounters.

Further empirical evidence reinforces these observations. Moore et al. (10) in a cohort of 766 arthroplasty patients, reported an 86% overall satisfaction rate with virtual follow-ups. Yet, rather than interpreting satisfaction purely as a subjective metric, this result may be understood as a proxy for engagement and compliance, reflecting patients’ perception of being continuously supported during recovery. Subsequent evidence suggests that telemedicine participation may be associated with improved adherence, fewer missed appointments, and earlier reporting of emerging concerns (11). These behavioral outcomes collectively highlight telemedicine’s potential to reshape patient–provider dynamics by fostering a sense of accessibility and shared responsibility.

Beyond convenience and compliance, telemedicine contributes to clinical quality assurance by enabling remote wound assessment and mobility tracking through digital imaging or wearable sensors.

Early pilot programs have demonstrated that high-resolution photo-based wound evaluation achieves high diagnostic concordance in selected settings compared with in-person examination, while mobile range-of-motion (ROM) applications allow real-time quantification of joint recovery trajectories (12). Moreover, recent evidence underscores that telemedicine not only improves logistical access but also enhances psychosocial support and patient engagement during recovery (13–15). These technologies may expand the amount of recovery-related information available to clinicians and allow patients to participate more actively in their own recovery.

However, the degree of success in telemonitoring depends on infrastructure, digital literacy, and system integration. Studies have noted variability in patient outcomes based on access to reliable internet, familiarity with technology, and the responsiveness of clinical teams to remote alerts (16). Thus, while telemedicine offers an elegant solution to logistical barriers, its efficacy hinges upon equitable digital access and institutional readiness.

Taken together, these findings suggest that telemedicine may represent an important extension of postoperative surveillance, supporting a more continuous and data-informed model of care. Its role in TJA recovery lies not only in extending the reach of clinicians but also in redefining what constitutes effective, patient-centered monitoring in the digital era.

### 2.2 Diagnostic and clinical comparability of telemedicine follow-ups

A critical question in evaluating telemedicine’s role in postoperative management is whether virtual assessments can achieve clinically acceptable diagnostic and follow-up outcomes compared with traditional in-person reviews. Existing evidence suggests potential comparability in selected domains, particularly for wound assessment accuracy, range-of-motion evaluation, and short-term complication detection, when video-based consultations and image-supported assessments are used in routine follow-up of uncomplicated TJA patients.

Moore et al. (10) provided complementary evidence from a patient-centered perspective, showing that high diagnostic confidence among clinicians corresponded to the 86% satisfaction rate reported by patients, emphasizing that clinical reliability translates directly into patient reassurance and trust. Fahey et al. (1) conducted a systematic review of telemedicine in orthopedic consultations across diverse musculoskeletal conditions. Although not limited to TJA populations, their findings provide important methodological support for remote diagnostic assessment–particularly in domains such as visual inspection, symptom evaluation, and decision-making concordance–which are directly applicable to routine postoperative follow-up after total hip and knee arthroplasty. This conclusion was reinforced by Windsor et al. (17), who analyzed over 1,200 teleconsultations and found that remote assessments showed high diagnostic agreement in selected orthopedic scenarios, particularly for wound integrity, joint swelling, and range-of-motion assessment. Together, these findings suggest that telemedicine may be capable of maintaining diagnostic accuracy in appropriately selected cases when appropriate imaging and communication protocols are followed.

From a functional recovery perspective, remote monitoring systems now employ structured outcome measures such as the Oxford Hip Score (OHS) and Knee Society Score (KSS) administered via digital platforms (18, 19). Studies utilizing these metrics have demonstrated comparable trajectories of recovery between telemedicine and conventional follow-ups, indicating that virtual care does not compromise objective rehabilitation progress. For instance, a multicenter randomized trial by Marsh et al. (20) compared postoperative outcomes between virtual and in-person cohorts after total knee arthroplasty and found no difference in complication rates, reoperation incidence, or range-of-motion recovery at 6 months. These data strengthen the argument that telemedicine provides a potentially comparable alternative in certain contexts, although results may vary depending on patient selection and study design.

Beyond objective indicators, the quality of clinical decision-making in telemedicine encounters has also been scrutinized. Importantly, disagreements occurred primarily in complex cases requiring palpation or imaging beyond standard two-dimensional photographs, suggesting that case selection is key to maintaining diagnostic parity. Furthermore, as imaging technology and data transmission improve, the diagnostic performance of telemedicine may continue to improve as imaging technology, data transmission, and standardized assessment protocols develop.

Collectively, these studies demonstrate that telemedicine follow-ups may provide diagnostically reliable and clinically acceptable alternatives to in-person care in selected scenarios–particularly for routine postoperative assessments involving wound inspection, symptom review, and functional monitoring in patients undergoing uncomplicated primary hip or knee arthroplasty. The convergence of high-resolution imaging, standardized digital assessment tools, and synchronous communication platforms has effectively bridged the diagnostic divide between remote and physical encounters. However, sustained comparability requires rigorous clinician training, standardized image capture protocols, and selective exclusion of patients with high-risk comorbidities or ambiguous symptoms.

A further limitation is that virtual assessment cannot fully reproduce key elements of an in-person orthopedic examination, including palpation, assessment of subtle warmth or effusion, gait observation under standardized conditions, and evaluation of complex pain generators (21, 22). Diagnostic reliability may also be affected by image and video quality, lighting, camera angle, patient positioning, internet bandwidth, and the ability of patients or caregivers to capture clinically useful wound or mobility images (23). Therefore, telemedicine follow-up should be supported by standardized image-capture instructions and clear escalation pathways for in-person assessment when symptoms are ambiguous or concerning.

### 2.3 Telerehabilitation and functional outcomes

Rehabilitation following TJA is a critical determinant of long-term outcomes, influencing pain reduction, range of motion, and functional independence. Traditional in-person physiotherapy, though effective, often imposes substantial logistical and financial burdens–including repeated hospital visits, transportation dependency, and limited access for patients in remote or mobility-restricted settings. Telerehabilitation, defined as the remote delivery of physical therapy and exercise supervision through digital communication platforms, has emerged as an evidence-based alternative that preserves clinical efficacy while expanding accessibility.

Early evidence from Moffet et al. (24) and Tousignant et al. (25) established that home-based telerehabilitation produced functional outcomes and patient satisfaction comparable to conventional physiotherapy. Their randomized controlled trials in total knee arthroplasty (TKA) patients demonstrated no significant difference in key indicators such as pain intensity, KSS, or 6-min walk test performance. These findings were further corroborated by a meta-analysis conducted by Wang et al. (26), which concluded that telerehabilitation may achieve outcomes comparable to standard outpatient physiotherapy in selected populations, with additional benefits in cost-effectiveness and patient adherence. These findings are consistent with earlier TJA-specific randomized trials, such as the study by Russell et al. (27), which demonstrated that internet-based telerehabilitation following total knee arthroplasty achieved functional outcomes comparable to conventional outpatient physiotherapy.

The mechanisms underlying these outcomes extend beyond mere convenience. Telerehabilitation promotes continuous patient engagement through personalized exercise tracking, progress feedback, and remote supervision by physiotherapists. Digital adherence tools–such as mobile reminders, wearable motion sensors, and video-guided sessions–have been shown to increase daily exercise compliance rates compared with standard home exercise programs (28). Moreover, the sense of autonomy afforded by self-managed, yet professionally supervised, recovery contributes to higher patient motivation and satisfaction. This reflects a broader movement toward patient engagement and self-management, where individuals actively participate in goal setting, progress monitoring, and recovery pacing.

From a psychosocial standpoint, telerehabilitation also mitigates feelings of isolation and anxiety commonly reported during the postoperative phase. Recent studies have found that virtual interaction with healthcare providers can maintain a sense of continuity and reassurance, especially among elderly patients recovering alone. For instance, Pastora-Bernal et al. (29) observed that patients enrolled in structured telerehabilitation programs after TKA reported significantly lower anxiety scores and higher self-efficacy, which correlated with faster achievement of functional milestones. These findings indicate that telerehabilitation not only restores physical function but also fosters psychological resilience and engagement.

Nevertheless, some challenges persist. Heterogeneity in digital literacy, lack of standardized exercise monitoring protocols, and unequal access to technology remain significant barriers to universal adoption. Certain patient subgroups–such as those with cognitive impairment or complex comorbidities–may require hybrid approaches that combine remote and in-person care. Future research should therefore focus on refining telemonitoring algorithms, integrating AI-driven motion analysis, and developing adaptive rehabilitation protocols that can personalize recovery intensity based on patient progress data.

In summary, telerehabilitation represents a potentially effective and patient-centered extension of postoperative care. By merging remote physiotherapy with digital monitoring and psychosocial support, it may achieve outcomes comparable to traditional rehabilitation–particularly in terms of pain reduction, functional scores, and mobility measures–in patients undergoing primary TKA who participate in structured, therapist-guided telerehabilitation programs delivered via video platforms or mobile applications. When integrated within comprehensive telemedicine pathways, telerehabilitation holds the potential to optimize functional outcomes, enhance engagement, and democratize access to high-quality orthopedic recovery services.

### 2.4 Interdisciplinary collaboration and continuity of care

One potential advantage of telemedicine in TJA lies in its ability to integrate multidisciplinary teams and sustain continuity of care across the postoperative trajectory. This advantage was particularly prominent during the COVID-19 pandemic. Gherman et al. (30) described the role of multidisciplinary telemedicine across diverse clinical settings. Although not specific to TJA, their findings highlight general principles of coordinated digital care that may be applicable to postoperative orthopedic pathways. Recovery after THA or TKA requires close coordination among surgeons, physiotherapists, nurses, and primary care providers to ensure optimal healing, prevent complications, and maintain adherence to rehabilitation regimens. Traditional in-person models often fragment this collaboration, as communication occurs episodically through separate appointments or delayed documentation. Telemedicine, by contrast, fosters a real-time, data-driven network of communication that unites multiple professionals around a shared digital care plan (31).

Kuether et al. (11) demonstrated that integrating teleconsultations into postoperative workflows resulted in higher patient compliance, faster reporting of complications, and more timely clinical interventions. Totty et al. (23) highlighted that early detection of wound abnormalities–such as erythema or excessive drainage–through photo-based telemonitoring allowed surgical teams to initiate prompt antibiotic or wound management interventions, reducing readmission rates. This proactive model of care contrasts with the reactive paradigm of traditional follow-up, where minor postoperative concerns often go unreported until they escalate.

Beyond surgical oversight, telemedicine platforms enable dynamic interdisciplinary exchanges between rehabilitation specialists and nursing staff. Physiotherapists can monitor range-of-motion progress and adapt exercise protocols based on real-time feedback (32), while nurses can assess pain levels, medication adherence, and psychological well-being through structured virtual check-ins (33). This bidirectional flow of information ensures that clinical decisions are contextualized within the patient’s overall recovery trajectory, rather than confined to isolated episodes of care. In this sense, telemedicine acts not only as a communication tool but as a coordination infrastructure that reinforces clinical continuity.

Recent studies further indicate that telemedicine enhances the psychosocial dimension of interdisciplinary care. Digital follow-ups provide consistent emotional reassurance, reduce anxiety, and strengthen the perceived therapeutic alliance between patients and their healthcare teams (34). For example, continuous virtual engagement with nurses and therapists has been associated with lower postoperative anxiety scores and greater adherence to self-care behaviors, particularly among elderly and socially isolated patients (35–37). These findings suggest that telemedicine serves as a psychosocial safety net–one that extends the reach of clinical empathy and emotional support beyond the hospital environment.

However, the success of interdisciplinary telemedicine depends on the integration of digital systems and professional workflows. Disparate electronic health record (EHR) systems, limited interoperability, and varying levels of digital literacy among providers remain major obstacles (38–40). Establishing standardized telehealth protocols, shared documentation platforms, and cross-disciplinary training programs is therefore essential to realizing telemedicine’s full potential. Moreover, equitable implementation requires addressing systemic disparities in digital access, ensuring that both patients and healthcare providers possess the tools and competencies necessary for effective participation.

In sum, telemedicine may help shift postoperative management from a fragmented, episodic model into a continuous, collaborative care ecosystem. By synchronizing multidisciplinary expertise through virtual coordination, it may support clinical communication, patient engagement, and continuity of care–shifting them from temporal proximity to sustained, networked engagement. This integrative framework is central to achieving equitable, patient-centered recovery pathways in contemporary orthopedic practice.

### 2.5 Psychological and social support in telemedicine recovery

While the physiological dimensions of recovery after TJA are well documented, the psychological and social aspects of postoperative healing are increasingly recognized as critical determinants of overall success. Pain, reduced mobility, and dependence on others during early recovery often contribute to anxiety, depressive symptoms, and a diminished sense of self-efficacy. Telemedicine, by sustaining continuous digital contact between patients and their care teams, has emerged as a pivotal means of addressing these psychosocial needs alongside clinical recovery. Psychological factors such as anxiety, pain catastrophizing, and self-efficacy have been shown to significantly influence postoperative outcomes after TJA, further underscoring the importance of addressing psychosocial dimensions during recovery (41, 42).

A growing body of evidence suggests that virtual follow-ups can substantially mitigate postoperative anxiety and enhance perceived support. Recent studies across a range of clinical contexts–including chronic disease management and non-orthopedic telehealth programs–have shown that patients participating in structured telemedicine interventions report lower anxiety and higher satisfaction (43–45). Although these findings are not specific to TJA populations, they provide relevant insights into the potential psychosocial benefits of sustained virtual engagement during recovery. These improvements stem largely from the immediacy and frequency of digital communication, which replaces the uncertainty of intermittent hospital visits with an ongoing sense of clinical presence. Even brief, asynchronous exchanges–such as message-based check-ins or remote symptom questionnaires–have been shown to reduce emotional distress by reinforcing the perception that professional help remains readily available.

Telemedicine also enhances the formation of a “therapeutic alliance,” a concept traditionally reserved for psychotherapeutic contexts but increasingly relevant in digital healthcare. The therapeutic alliance refers to the relational bond and mutual trust between patient and clinician, which strongly predicts adherence to treatment and perceived recovery quality (46, 47). Through virtual consultations, video check-ins, and personalized feedback loops, telemedicine fosters a sense of partnership and shared responsibility that strengthens patient motivation. For instance, Ali et al. reported that patients receiving video-based telerehabilitation exhibited significantly higher scores on the Working Alliance Inventory (WAI) (48), indicating deeper relational engagement and trust in their providers compared with those in standard follow-up care.

Beyond the patient–clinician dyad, telemedicine fosters community-level connectedness through group-based virtual rehabilitation and peer-support forums (49). Such digital environments provide opportunities for patients to share experiences, exchange coping strategies, and normalize their postoperative challenges–an aspect particularly beneficial for elderly individuals or those living alone. Studies have shown that group tele-sessions improve not only psychological resilience but also adherence to home exercise programs by leveraging social accountability (50, 51). In this sense, telemedicine transforms recovery from an isolated medical process into a socially supported experience of shared progress.

However, psychological benefits are not uniformly distributed. Patients with limited digital literacy, cognitive impairment, or pre-existing anxiety disorders may derive less reassurance from virtual interactions, and in some cases, the absence of physical presence can exacerbate uncertainty. To address these disparities, hybrid care models–combining periodic in-person visits with ongoing digital touchpoints–have been recommended as a strategy to balance emotional reassurance with technological accessibility (52, 53). Moreover, incorporating behavioral health professionals into telemedicine pathways can further personalize support, integrating cognitive-behavioral interventions and motivational coaching directly into orthopedic follow-ups.

In sum, the psychological and social dimensions of telemedicine recovery extend beyond patient satisfaction to encompass emotional security, therapeutic trust, and social engagement. By maintaining continuous contact, promoting shared accountability, and integrating mental health awareness into digital care frameworks, telemedicine may contribute to a more holistic model of postoperative recovery–one that aligns physiological healing with psychological resilience and social connectedness.

### 2.6 Challenges and future directions

Despite growing evidence supporting telemedicine’s clinical efficacy and patient acceptance in TJA, several systemic, technical, and ethical challenges continue to hinder its universal implementation. These challenges highlight the need for a balanced perspective that recognizes both the transformative potential and the inherent limitations of digital healthcare in postoperative management.

#### 2.6.1 Digital divide and equity of access

One of the most pressing barriers remains the unequal distribution of digital infrastructure, device availability, internet connectivity, and digital literacy, particularly among elderly, rural, socioeconomically disadvantaged, and cognitively impaired patients (54). Although telemedicine may reduce travel-related barriers after TJA, it can also create new forms of exclusion for patients who lack smartphones, stable broadband access, caregiver support, or confidence in using digital platforms (55). This issue is especially relevant in arthroplasty populations, where many patients are older adults and may require assistance with wound photography, video consultations, or app-based rehabilitation monitoring. Therefore, telemedicine should not be implemented as a universal replacement for in-person follow-up. Instead, hybrid models, caregiver-assisted workflows, simplified user interfaces, and targeted digital literacy support are needed to ensure that remote care expands rather than restricts access.

#### 2.6.2 Data privacy, security, and ethical considerations

As telemedicine platforms collect sensitive clinical and behavioral data, concerns over privacy, data ownership, and cybersecurity have become increasingly salient. Breaches or misuse of digital health information can erode patient trust and undermine engagement. Regulatory frameworks such as the Health Insurance Portability and Accountability Act (HIPAA) in the United States and the General Data Protection Regulation (GDPR) in Europe provide baseline safeguards, yet interpretation and enforcement in telehealth contexts remain inconsistent (56, 57). Future policies must therefore address issues of cross-border data sharing, informed consent in virtual environments, and ethical governance of AI-assisted monitoring systems.

#### 2.6.3 Clinical validation, staff workload, and system integration

Although accumulating evidence suggests that telemedicine can be clinically acceptable in selected TJA follow-up scenarios, standardization of telehealth assessment protocols remains limited. Variability in image quality, device calibration, patient positioning, and clinician interpretation can introduce diagnostic uncertainty, particularly in complex cases. In addition, remote care does not automatically reduce workload for clinical teams. Asynchronous messages, wound photographs, app-generated alerts, and rehabilitation data streams may increase the volume of information requiring review, triage, documentation, and follow-up. Without adequate staffing, role allocation, and escalation protocols, telemedicine may shift rather than reduce the burden of postoperative care.

Integration with existing hospital information systems also remains a major challenge. Fragmented electronic health records, non-interoperable telehealth platforms, and separate rehabilitation databases can disrupt continuity and create duplicated documentation. For telemedicine to be safely embedded into TJA pathways, institutions need shared documentation systems, defined responsibilities among surgeons, nurses, and physiotherapists, and clear protocols for responding to abnormal findings or patient-reported concerns.

#### 2.6.4 Reimbursement, cost-effectiveness, and policy alignment

The sustainability of telemedicine depends heavily on reimbursement structures, institutional incentives, and real-world cost-effectiveness. Although virtual follow-up may reduce travel costs, time away from work, and unnecessary clinic visits for patients, these savings must be weighed against the costs of digital platforms, technical support, staff training, data security, workflow redesign, and clinician time spent reviewing asynchronous information. Current evidence on cost-effectiveness in TJA remains limited and may vary substantially across healthcare systems, reimbursement models, and patient populations. Temporary expansions in telehealth billing during the COVID-19 pandemic catalyzed adoption (58), but many policies remain provisional. Establishing sustainable reimbursement models that recognize virtual follow-ups, asynchronous communication, and remote physiotherapy is critical to long-term implementation.

#### 2.6.5 Emerging innovations and future research

Looking forward, telemedicine is poised to evolve through integration with artificial intelligence (AI), wearable biosensors, and extended reality technologies. Machine learning algorithms have already demonstrated promise in automating wound detection, predicting infection risk, and personalizing rehabilitation intensity based on motion data. Virtual reality (VR) and augmented reality (AR) platforms are being explored for immersive rehabilitation, allowing patients to perform guided exercises in interactive digital environments that simulate real-world movement patterns (59). These technologies hold potential not only for improving functional outcomes but also for enhancing motivation and engagement.

Despite encouraging short-term findings, the long-term effectiveness of telemedicine after TJA remains uncertain. Most available studies focus on early postoperative outcomes such as satisfaction, wound monitoring, range of motion, or short-term functional recovery. Less is known about whether telemedicine affects long-term implant-related outcomes, late complications, revision rates, sustained rehabilitation adherence, health service utilization, or patient-reported quality of life beyond the early recovery period. Equally important is the call for longitudinal, multicenter studies that examine the durability of telemedicine outcomes beyond the early postoperative phase. Future research should explore how digital continuity affects long-term implant survival, revision rates, and cost-effectiveness across diverse healthcare settings. Ethnographic and qualitative investigations may further elucidate how patients perceive telemedicine as part of their identity reconstruction and social reintegration after surgery–a dimension often overlooked in quantitative studies.

Ultimately, the trajectory of telemedicine in orthopedic care must balance technological innovation with human-centered design and policy foresight. The goal is not merely to digitize existing workflows but to reimagine postoperative recovery as a holistic, interconnected process that integrates physical, psychological, and social dimensions. Achieving this vision will require collaborative governance among clinicians, technologists, policymakers, and patients, supporting the responsible and equitable integration of telemedicine into postoperative orthopedic care.

## 3 Conclusion

Telemedicine has emerged as an increasingly important component of postoperative care in total joint arthroplasty, particularly following the expansion of digital health during the COVID-19 pandemic. Current evidence suggests that virtual follow-up, remote wound monitoring, and telerehabilitation may support outcomes comparable to conventional in-person care–especially in terms of wound monitoring, early functional recovery, and patient satisfaction–when implemented in structured modalities and in appropriately selected patients with uncomplicated postoperative courses. By reducing geographical, mobility-related, and logistical barriers, telemedicine may contribute to more continuous and patient-centered recovery pathways, particularly for individuals who face difficulties attending repeated hospital visits.

Importantly, the role of telemedicine in TJA should not be interpreted as a replacement for face-to-face care, but rather as a complementary approach. Remote monitoring technologies, digital rehabilitation platforms, and virtual consultations may help extend clinical observation beyond the hospital setting, facilitate earlier recognition of selected complications, and support communication between patients and multidisciplinary care teams. In this context, telemedicine may contribute to a more proactive and coordinated model of postoperative management.

Nevertheless, this potential should be interpreted cautiously. Telemedicine cannot fully replace the diagnostic value of hands-on physical examination, and the reliability of remote assessment may be affected by image or video quality, patient positioning, connectivity, and the ability of patients or caregivers to provide clinically useful information. The current evidence base also remains heterogeneous, with differences in study design, patient populations, digital platforms, follow-up duration, and outcome measures. In addition, digital exclusion, staff workload, platform interoperability, data governance, reimbursement, and real-world cost-effectiveness remain unresolved challenges. These limitations suggest that telemedicine should be implemented selectively and within well-defined clinical pathways, with appropriate patient selection, adequate staffing, and timely access to in-person evaluation when needed.

Future research should prioritize well-designed, multicenter, and longitudinal studies to better understand the long-term effectiveness, safety, cost-effectiveness, and equity implications of telemedicine in TJA. Emerging technologies, including wearable sensors, artificial intelligence, and immersive rehabilitation platforms, may further expand the possibilities of remote postoperative care, although their clinical value requires rigorous validation before widespread adoption. Overall, telemedicine may serve as a valuable adjunct to traditional postoperative care, with its greatest benefit likely arising from thoughtful integration into hybrid, patient-centered models rather than from replacing conventional orthopedic follow-up entirely.
